# Transcriptome Profiling Reveals Enhanced Mitochondrial Activity as a Cold Adaptive Strategy to Hypothermia in Zebrafish Muscle

**DOI:** 10.3390/cells12101366

**Published:** 2023-05-11

**Authors:** Thomas Cahill, Sherine Chan, Ian M. Overton, Gary Hardiman

**Affiliations:** 1 School of Biological Sciences, Institute for Global Food Security, Queen’s University Belfast, Belfast BT9 5DL, UK; tcahill01@qub.ac.uk; 2 Department of Drug Discovery and Biomedical Sciences, Medical University of South Carolina, Charleston, SC 29425, USA; sherine@neuroenetherapeutics.com; 3 JLABS at the Children’s National Research and Innovation Campus, Washington, DC 20012, USA; 4 Patrick G Johnston Centre for Cancer Research, Queen’s University Belfast, Belfast BT9 7AE, UK; i.overton@qub.ac.uk

**Keywords:** zebrafish, transcriptomics, muscle, radiation, induced torpor, DNA damage, mitochondria, hibernation

## Abstract

The utilisation of synthetic torpor for interplanetary travel once seemed farfetched. However, mounting evidence points to torpor-induced protective benefits from the main hazards of space travel, namely, exposure to radiation and microgravity. To determine the radio-protective effects of an induced torpor-like state we exploited the ectothermic nature of the *Danio rerio* (zebrafish) in reducing their body temperatures to replicate the hypothermic states seen during natural torpor. We also administered melatonin as a sedative to reduce physical activity. Zebrafish were then exposed to low-dose radiation (0.3 Gy) to simulate radiation exposure on long-term space missions. Transcriptomic analysis found that radiation exposure led to an upregulation of inflammatory and immune signatures and a differentiation and regeneration phenotype driven by *STAT3* and *MYOD1* transcription factors. In addition, DNA repair processes were downregulated in the muscle two days’ post-irradiation. The effects of hypothermia led to an increase in mitochondrial translation including genes involved in oxidative phosphorylation and a downregulation of extracellular matrix and developmental genes. Upon radiation exposure, increases in endoplasmic reticulum stress genes were observed in a torpor+radiation group with downregulation of immune-related and ECM genes. Exposing hypothermic zebrafish to radiation also resulted in a downregulation of ECM and developmental genes however, immune/inflammatory related pathways were downregulated in contrast to that observed in the radiation only group. A cross-species comparison was performed with the muscle of hibernating *Ursus arctos horribilis* (brown bear) to define shared mechanisms of cold tolerance. Shared responses show an upregulation of protein translation and metabolism of amino acids, as well as a hypoxia response with the shared downregulation of glycolysis, ECM, and developmental genes.

## 1. Introduction

In humans, skeletal muscle is made up of myofibres that each contain several myofibrils that in turn are made up of sarcomeres. Sarcomeres are the basic contractile unit of the muscle consisting of actin and myosin protein filaments that utilize ATP to contract and generate force for movement. Fascicles are made up of bundles of myofibres, held together by the extracellular matrix (ECM) that make up the muscle tissue, which is highly vascularized and innervated. Muscle fibres can be classified into slow twitch type I fibres or fast twitch type IIA and type IIX fibres, often identified by their myosin heavy chain isoforms, containing MHCI, MHCIIa, and MHCIIX, respectively [1]. Slow twitch type I fibres have a high oxidative capacity and low glycolytic capacity and contain higher levels of mitochondria that enable longer periods of force generation and fatigue resistance. Fast twitch fibres have a faster, stronger contraction but are quicker to fatigue. Type IIA fibres exhibit high oxidative capacity and intermediate glycolytic capacity, whereas type IIX have a high glycolytic capacity and low oxidative capacity [2]. Muscle tissue is a dynamic organ whose mass can be influenced by physical activity, nutritional status, or disease by modifying the balance between protein synthesis and degradation, leading to measurable changes in muscle fibre size and number, as well as influencing fibre development or leading to fibre type switching. Key regulators of muscle hypertrophy include IGF-1, and testosterone which increase protein synthesis through the Akt-mTor signaling pathway [3,4]. Conversely, muscle atrophy is associated with the activation of the ubiquitin proteasome system that mediates protein degradation, as well as the expression of the atrogenes *MuRF1*, and *MAFbx*, which are activated by FoxO and Smad transcription factors [5]. Anabolic stimuli such as high endurance exercise including running or cycling, for example, have shown associations with increased numbers of type I fibres, whereas high-velocity weight training can cause fibre type shifting to type IIA fibres from type I fibres [2,6]. Conversely, inactivity, bed rest, sarcopenia, and myopathies can negatively influence muscle quality. Sarcopenia, for instance, is characterized by muscle loss in the aging population that affects muscle strength and power and can lead to mobility issues. It is further characterized by a reduced number of satellite cells which play a key role in muscle regeneration, changes to the structure of the sarcoplasmic reticulum, and a loss of mitochondria [7].

Spaceflight poses challenges to muscle physiology through exposure to microgravity, galactic cosmic rays/solar particle events, or the living environment that can contribute to or influence a decline in muscle quality. Microgravity might be the most significant of these variables by facilitating unloading, leading to a well-documented atrophy similar to that induced by inactivity [8]. In fact, early spaceflight missions utilising rats found that spaceflight exposure as short as 4 days led to a decrease in muscle mass. They also found that anti-gravity muscles were particularly affected in this time-frame, along with evidence of slow twitch fibres expressing increased levels of fast myosin heavy chain, suggestive of a slow-to-fast twitch conversion [9]. Results were corroborated in studies of short-term spaceflight (5–11 days), where human vastus lateralis muscles showed fewer type I fibres, reduced fibre cross-sectional areas, increased glycolytic enzymes in type I fibres, and fewer capillaries per fibre [10]. However, further studies revealed a larger impact on type II fibres in longer-duration missions [11]. Such structural changes negatively impact functionality, as short-duration missions can decrease knee extension torque by 12%, increasing to 31% in longer-duration missions [12]. Observations into the mechanisms of reduced muscle mass during spaceflight revealed a translational deficit leading to a reduction in protein synthesis [13]. Studies also show a downregulation of focal adhesions mediating fibre-matrix interactions, and increased antioxidant response in slow twitch fibres, indicating increased oxidative stress [14]. Ground models that have been used to dissect the effects of unloading and radiation have demonstrated that radiation does not sensitise muscle to unloading-related atrophy [15], although, it has been shown that low-dose radiation can lead to muscle remodelling that could impact repair [16]. Similarly, chronic low-dose radiation was found to impact the satellite cells, which is important for muscle regeneration [17].

Current countermeasures against muscle atrophy during space flight entail a 2 h daily resistive exercise regime and while it is generally considered effective, deficits in muscle mass are still observed [18]. Additionally, shielding and minimising time spent in spaceflight remain the only two effective countermeasures for radiation exposure. When it comes to long-term interplanetary space travel, the idea of induced torpor as a countermeasure to the effects of spaceflight has been the topic of much discussion given the evidence that it can provide radio-protective effects [19]; furthermore, while undergoing long periods of inactivity and fasting, muscles of hibernating animals are more resistant to atrophy [20]. The bear represents an excellent model for long-term torpor, due to modest reductions in basal metabolic rate, comparable body mass, and its utilisation of torpor for 5–7 months at a time, comparable to a journey to Mars. Research shows that muscles in black bears resist atrophy through increased ribosome and protein biosynthesis with a downregulation of glycolysis while hibernating [21]. Further studies reveal that bears have the ability to recycle nitrogen from reabsorbed urea, providing a more favourable nitrogen balance for protein synthesis [22]. Moreover, one study showed that atrophy resistance may be conferred through the downregulation of atrophy-related TGF-β signalling and an increase in growth-related BMP signalling pathways.

Zebrafish (*Danio rerio*) represent an excellent model for the study of human disease due to their small size, fast reproductive rates, and ease of husbandry. The human genome shares ~70% of its protein-coding genes with zebrafish and 82% of genes associated with human disease have a zebrafish orthologue [23]. In addition, 86% of 1318 human drug targets have a zebrafish orthologue, making zebrafish an excellent model for characterising the effects of pharmaceutical interventions [24]. Hence, zebrafish models have been used to characterise the basic biology of muscle stem cell formation and maintenance as well as to provide insights on the pathology of muscular dystrophies [25]. In addition, their ectothermic natures enable a low-fidelity model for the study of hypothermic states in comparison to controls as their body temperatures and metabolisms can be easily manipulated with control of their ambient temperatures. We, therefore, investigated the value of zebrafish in replicating the hypothermic state seen in hibernating animals through a cross-species comparison with the hibernating *Ursus arctos horribilis* (brown bear), defining any shared mechanisms of cold tolerance in the muscle; additionally, investigating whether the hypothermic state in zebrafish offered radio-protective effects. We exploited a zebrafish model previously described [26] to lower the metabolism and activity of the zebrafish and performed transcriptomic analysis after exposure to 0.3 Gy of radiation, similar to that which would be experienced by astronauts on a journey to Mars.

## 2. Materials and Methods

### 2.1. Zebrafish Husbandry

Experiments were carried out with adult AB Danio rerio (zebrafish), obtained from the Zebrafish International Resource Center. Adult zebrafish were housed 6 fish per liter of beaker in a 28.5 °C incubator with a 14-h on (bright) and 10-h off (dark) light cycle. They were cared for and bred according to standard breeding practices. A standard Gemma Micro 300 diet (Skretting, Westbrook, ME, USA) was administered every other day. To maintain water viability, any remaining debris was aspirated 20 min after feeding and 75% of the water was replaced with reservoir water daily with the addition of sodium bicarbonate, instant ocean salt, and stress coat to reverse osmosis water to maintain a pH of 7.4. In addition, all beakers that housed the zebrafish were washed and autoclaved prior to use. All procedures were performed under the Institutional Animal Care and Use Committee (IACUC) guidelines (IACUC-2018-00278) of the Medical University of South Carolina (MUSC). All animals were treated humanely and with consideration for relieving suffering.

### 2.2. Development of the Induced Torpor Model and Radiation Protocol

**Figure 1:**
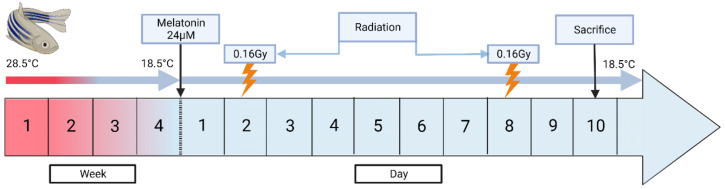
A schematic diagram showing the establishment of the torpor+radiation group (18.5-mel-rad). This group was acclimated at 18.5 °C from 28.5 °C, over a 4-week period to avoid thermal shock. They were administered 24 µM on the start of the experimental timeline, with radiation exposure of 16.34 cGy occurring on the 2nd and 8th days of the timeline for a total of wholebody dose of 32.68 cGy. The fish were then sacrificed on the 10th day.

As shown in Table 1, several experimental groups were used to evaluate induced torpor as a radiation countermeasure. We first established a melatonin group (2.8.5-mel) by administering 24 µM melatonin for 10 days to a group of zebrafish maintained at 28.5 °C, which has been cited as an optimal breeding temperature and has been used as a standard temperature for developmental studies [27]. A mixture of melatonin and salt water was added daily to maintain melatonin levels after changing of 75% of water (Cavallo and Hassan, 1995). Melatonin was purchased from Sigma-Aldrich (St. Louis, MI, USA) with a purity of 98% or higher, which was stored in powder form at −20 °C, and dissolved in DMSO before use. A reduced temperature (18.5-Ctrl) group was acclimated to 18.5 °C to constitute a 10 °C reduction in body temperature, over a 4-week period with decrements of 2.5 °C, which has previously been shown to avoid thermal shock [28]. Malek et al., (2004) also demonstrated a successful year-long maintenance of zebrafish acclimated to 18 °C from 28 °C [28]. Similarly, an ‘induced torpor’ group (18.5-mel) was acclimatised to 18.5 °C before administration of 24 µM melatonin for 10 days. We also established a low-dose radiation (28.5 rad) group that were maintained at 28.5 °C and exposed to a total whole-body dose of 32.68 cGy. Radiation exposure occurred at 163.40 cGy /min for 6 s, with a total exposure of 16.34 cGy on the 2nd and 8th days of the experimental timeline. Before radiation exposure, zebrafish were anesthetized with 0.02% tricaine, placed individually in 60 mm × 15 mm Petri dishes in water, and on 3-inch spacers ready for irradiation. Irradiation was performed at MUSC according to IACUC-2018-00278 using a Shepherd model 14,368 irradiator with a cesium-137 radiation source, serial number 8020 (JL Shepherd and Associates, San Fernando, CA, USA). After radiation exposure, the fish were placed in a temporary tank free of tricaine for recovery and returned to the main tank with other fish from the same experimental group. An ‘induced torpor+radiation’ group (18.5-mel-rad) was also set up as shown in Figure 1, where fish were acclimated to 18.5 °C, over a 4-week period, treated with melatonin at the start of the 10-day experimental timeline, and exposed to radiation on the 2nd and 8th days of the timeline. Finally, a control group (28.5-Ctrl) were maintained at an ambient temperature of 28.5 °C without the addition of melatonin or exposure to radiation. All fish were sacrificed on the 10th day of the experimental timeline

### 2.3. RNA Extraction and Sequencing

**Figure 2:**
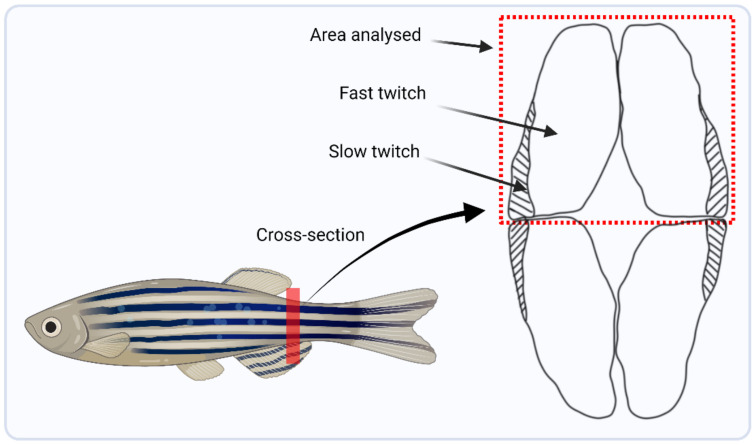
Schematic diagram highlighting the trunk location from where muscle was harvested, marked by the red line, and a lateral cross-sectional area showing the dorsal region of muscle that was dissected for RNA and DNA extraction for downstream analysis.

A section of dorsal muscle was dissected from the zebrafish highlighted in Figure 2 which consists primarily of fast twitch muscle fibres [29]. The miRNeasy Mini kit (Qiagen, Hilden, Germany) was used to extract total mRNA from the muscle. Next, the mRNA seq kit (poly A capture, KAPA RNA hyper) was used to prepare mRNA libraries (Roche, Indianapolis, IN, USA), taking 100 ng of total RNA from the muscle and following the manufacturer’s protocol. RNA sequencing (RNAseq) was performed at the Queen’s University Belfast, Genomics Core Technology Unit on an Illumina Next SEQ 2000 instrument, and sequenced to a minimum depth of 50 million reads using a forward stranded PE50 strategy.

### 2.4. RNA-Seq Data Processing and Differential Gene Expression

**Figure 3:**
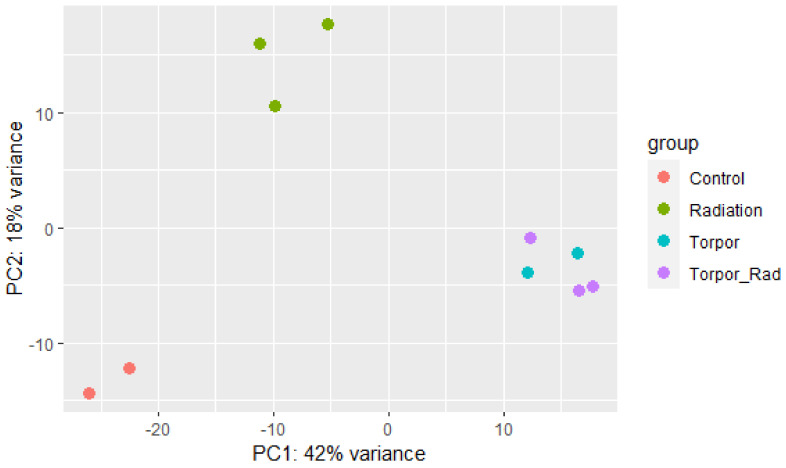
Principle component analysis plot showing samples of each experimental group clustering based on similarity. The plot shows distinct clustering between the control group (red), the radiation group (green), and the torpor (blue) and torpor+radiation (purple) groups which are clustered together, suggesting that the effects of torpor are more dominant than radiation exposure.

Read quality was assessed using FastQC [30]. STAR aligner [31] aligned reads to the zebrafish genome (GRCz11) and HTSeq [32] extracted read counts per transcript. DESeq2 [33] was used to determine differentially expressed genes (DEGs) in experimental groups versus the control group. A principal component analysis (PCA) plot was generated to show the clustering of samples based on similarity (Figure 3). An FDR-adjusted *p*-value was applied to DEGs for use in downstream analysis, calculated using the Benjamini-Hochberg procedure [34]. Ensembl human orthology [35] was utilised to append human orthologues to analogous zebrafish gene IDs, to take advantage of the better annotation of human genes, as established by Huff et al., [36]. In order to validate our model of induced torpor in the zebrafish we compared it to torpor utilised naturally by *Ursus arctos horribilis* (grizzly bear). We obtained transcriptomic data from the muscle of six active and hibernating bears from Genbank BioProject PRJNA413091 and generated DEGs following the pipeline outlined above.

### 2.5. Pathway Analysis

Over-representation analysis (ORA) was carried out on up or downregulated human orthologue DEGs using ToppFunn [37] to define enriched biological processes and KEGG pathways. Similarly, iPathwayGuide (Advaita Bioinformatics, Ann Arbor, MI, USA) [38] was used to perform pathway impact analysis, which utilises pathway topology to consider the positions and interactions between genes in each pathway as well as the type of interaction and the function of each gene, to help reduce false positives. STRING [39] was used to generate protein interaction networks, and g:Profiler [40] and EnrichmentMap [41] determined gene-set clusters using Cytoscape v3.9.1 [42].

### 2.6. DNA Damage Assay

DNA was extracted from the muscle of each experimental group including the control using the QIAamp DNA Micro Kit according to the protocol. DNA was then digested using nuclease P1 according to the manufacturer’s instructions and the pH was adjusted to 7.5–8.5 using 1M Tris. The levels of 8-hydroxy-2-deoxyguanosine (8-OHdG) were measured using the 8-OHdG ELISA kit (ab201734, Abcam) following manufacturer’s instructions. Absorbance values were measured using a CLARIOstar microplate reader and a standard curve was used to determine 8-OHdG concentrations. F-tests were performed to test for equal variance within populations and t-tests were performed to test for significance.

### 2.7. Gene Expression Validation

A reverse transcriptase reaction was set-up using a concentration of 0.1 µg of RNA constituted in 20 µL of nuclease-free water from 2 biological replicates in: 28.5-ctrl, 28.5-rad, 18.5-mel, 18.5-mel-rad. To synthesise cDNA, 1 µL of iScript Reverse transcriptase was added to the reaction tube along with 4 µL of iScript Reverse Transcription Supermix (Bio-Rad) and samples were placed in a thermocycler following the protocol in Table S1. The resulting cDNA (2.4 ng, 2.4 µL) was used in the qPCR reactions in quantifying gene expression with the addition of exon spanning primers (1.6 µL of forward and reverse primers) designed using Primer-Blast [43] and shown in Table S2. Next, 10 µL of SYBR Green qPCR was added to the reaction tube (ThermoFisher, Waltham, MA, USA) and 6 µL of nuclease-free water was added to make a final volume of 20 µL. The plate was assayed using a Roche LightCycler 480 Instrument II (Roche Diagnostics, Rotkreuz, Switzerland) using the protocol described in Table S3. The relative mRNA expression was determined against *actn2b* and *gapdh* reference genes for normalisation and values for the experimental groups (28.5-rad, 18.5-mel, 18.5-mel-rad) were compared with values from the control group (28.5-ctrl). Finally, T-tests were used to test for significance and the results were plotted using GraphPad Prism 8.4.3 (San Diego, CA, USA).

## 3. Results

### 3.1. Exposure to Low Dose Radiation in the Muscle Decreases DNA Repair Processes

**Figure 4:**
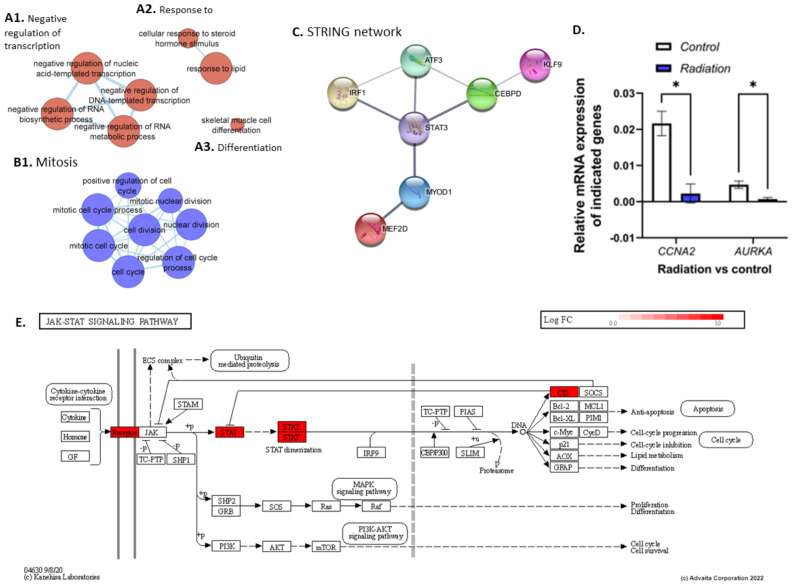
Clusters (**A**,**B**) were generated using EnrichmentMap and up (red) and downregulated (blue) genes, respectively. Cluster (**A1**) shows GO terms related to the negative regulation of transcription. (**A2**) shows a response to steroid hormone stimulus and lipids while (**A3**) shows skeletal muscle cell differentiation. (**B1**) shows downregulation of GO terms related to cell division. (**C**) shows a string network generated using the DNA transcription factor activity GO term (GO:0001216), showing protein interaction between STAT3 and MYOD1. (**D**) shows results from the qPCR analysis which shows significant downregulation of *CCNA2* and *AURKA* in the radiation group compared with controls (28.5-rad vs. 28.5-Ctrl). (**E**) shows genes upregulated in the JAK/STAT pathway in the radiation vs control group. * *p* < 0.05.

To define the impact of low-dose radiation in fast twitch muscle we performed differential expression analysis (DEA) of the radiation group versus the control group (28.5-rad vs. 28.5-Ctrl). It revealed an upregulation of 50 DEGs and downregulation of 99 DEGs (*q* ≤ 0.4) (Table S4) that were subject to ORA and impact analysis (Tables S5 and S6). ORA of the *upregulated* genes returned gene-sets related to DNA transcription factor activity (GO:0140297, GO:0001216) involving transcription factors with roles in stress response (*AFT3*) [44], immune response (*IRF1*, *CEBPD*) [45,46], and differentiation and regeneration (*MYOD1*, *STAT3*, *MEF2D*) [47,48,49]. A protein interaction network (PPI) was generated using genes in the transcription factor activity gene ontology (GO) term (GO:0001216), revealing a protein interaction between *STAT3* and *MYOD1* (Figure 4C). Furthermore, gene-sets related to the phenotypes controlled by the transcription factors were enriched, namely, response to steroid hormone signalling (GO:0071383, GO:0032870) (Figure 4A2) and the pro-inflammatory NF-kappaB and IL-6 signalling pathways (137932, M183). Additionally, muscle cell development, differentiation and cell fate determination, as well as muscle cell proliferation (GO:0007517, GO:0035914 GO:0001709, GO:1904707) were enriched (Figure 4A3), indicating muscle remodelling in response to insult. This was accompanied by upregulation of myosin filament assembly and organisation (GO:0031034 GO:0031033). Enrichment of metabolic genes suggests changes to organic and carboxylic acid metabolism (GO:0006082 GO:0019752), as well as the negative regulation of RNA metabolic processes (GO:0051253), while anabolic genes relating to protein translation were also enriched (GO:0006418, GO:0043038).

Surprisingly, ORA of *downregulated* genes revealed enrichment of a response to DNA damage and DNA damage checkpoint signalling (GO:0042770 GO:0044774), as was also true for DNA Double-Strand Break Repair (1309095) and DNA Repair pathways (1270350) in which genes such as *EME1*, *PRKDC*, and *RAD51C* were downregulated. In addition, genes related to telomere maintenance were downregulated (GO:0000723 GO:0016233 GO:0032200). Several cell cycle/division GO terms were also downregulated (GO:0000278 GO:0000279 GO:0051301) (Figure 4B1), as were proliferative pathways such as the Aurora B and FOXM1 signalling pathways (138080, M176) involving genes such as *CCNA2*, *CCNB1*, *CCNB2*, and *AURKA*. qPCR analysis of *AURKA* (*p* = 0.004) and *CCNA2* (*p* = 0.02) showed a significant downregulation in comparison to the control group (28.5-Ctrl)(Figure 4D). The cytoskeleton, which plays an important role in morphology and cell division, was perturbed, with enrichment of microtubule and spindle organisation genes (GO:0000226 GO:0007051). Also enriched using the downregulated genes were terms related to the regulation of ubiquitin protein ligase activity (GO:1904666), accompanied by the positive regulation of both protein metabolism and translation (GO:0032270, GO:0045727). Moreover, the results show decreased fibroblast proliferation (GO:0048144), as well as genes connected with oogenesis and oocyte development, suggesting changes in female reproductive processes. An EnrichmentMap was generated using up and downregulated genes, shown in Supplementary Figure S1.

### 3.2. Induced Torpor in Zebrafish Leads to Increased Mitochondrial Gene Expression in Zebrafish Muscle

**Figure 5:**
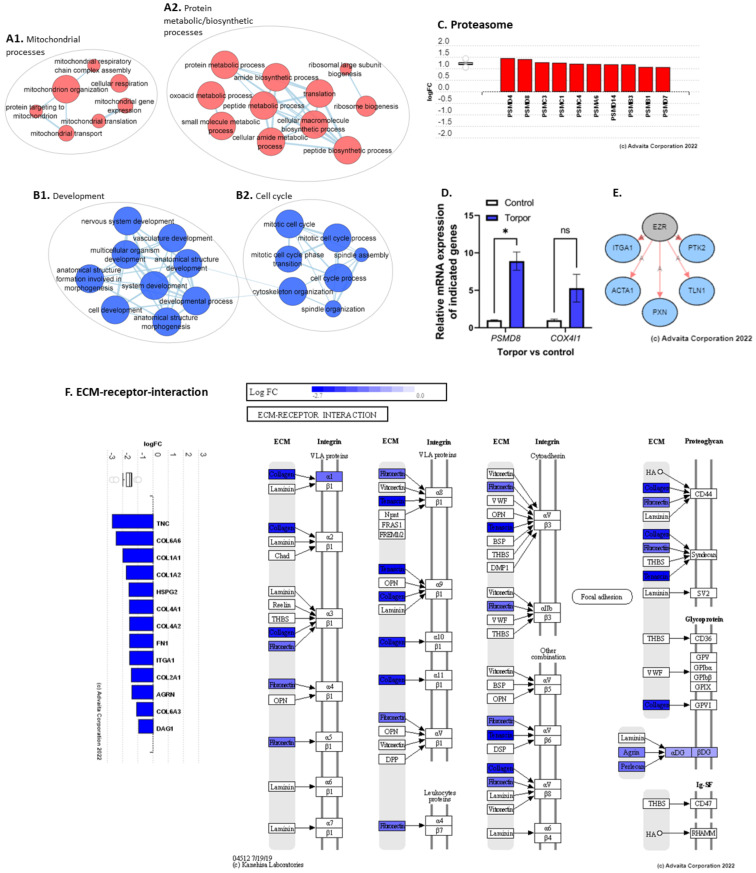
(**A1**) shows the upregulation of mitochondrial processes. (**A2**) shows the upregulation of GO terms involved in both protein metabolism and biosynthesis. (**B1**,**B2**) show the downregulation of developmental and cell cycle terms, respectively. (**C**) shows the upregulation of proteasomal subunit genes. (**D**) shows the qPCR analysis showing a significant upregulation of *PSM8* and a non-significant upregulation of *COX4I1.* (**E**) shows the predicted inhibition of upstream regulator EZR. (**F**) shows the downregulation of genes involved in the ECM such as collagens, integrins, and fibronectins. * *p* < 0.05, ns = non-significant.

To determine how temperature+melatonin (induced torpor) affects the muscle transcriptome, DEA of the torpor group vs control (18.5-mel vs. 28.5-Ctrl) was performed revealing the upregulation of 469 and downregulation of 271 DEGs (*q* ≤ 0.1) (Table S7) that were subject to ORA and impact analysis (Tables S8 and S9). In contrast to the previously reported decrease in expression of metabolic genes in the gastrointestinal tract and liver [26,50], ORA of upregulated genes enriched metabolic processes in the muscle, such as cellular respiration (GO:0045333), electron transport chain (ETC) (GO:0022900), and oxidative phosphorylation (GO:0006119) involving *SDHC*, *COX4I1*, and *COX11*. qPCR analysis of *COX4I1* expression found a non-significant (*p* = 0.08) increase in comparison to controls (18.5-mel vs. 28.5-Ctrl) (Figure 5D). Also upregulated were mitochondrial gene expression (GO:0140053), and translation (GO:0032543) (Figure 5A1), involving *TFAM*, and *MRPL51*, respectively. We know that muscle mass is affected by an imbalance in protein synthesis and degradation. Despite the reduced activity, we report both an upregulation of several ribosome genes (GO:0042254), supplemented by biosynthesis of amino acid pathways involving the *ASNS* gene, pointing to increased protein synthesis; as well as proteasomal degradation pathways (M10680) involving *PSMD4*, *PSMD8*, and *PSMC3*, (Figure 5C), along with amine, and aspartate metabolism (GO:0009308, GO:0009066). In agreement, qPCR analysis of *PSMD8* found a significant (*p* = 0.01) increase in expression (Figure 5D). It has been established that ketones act as an alternative energy source in response to fasting [51], in cold-adapted rats [52], and in the hearts and brains of hibernating ground squirrels (*Spermophilus beldingi*) [53]. Here, we also report increased ketone metabolism in the cold-acclimated zebrafish (GO:0042180). Additionally, cold acclimation at 18 °C significantly reduces oxygen consumption in zebrafish [54], consistent with enrichment of the hypoxia response (GO:0001666). Hypoxia has been shown to generate reactive oxygen species (ROS) in skeletal muscle, which could explain the enrichment of oxidative stress (GO:0006457) and the S-adenosylmethionine metabolic processes (GO:0046500) that regulate glutathione synthesis. In addition, the enrichment of pathways related to the DNA damage response (1269744), regulation of cell cycle (GO:1901987), and the regulation of apoptosis (1270295), are characteristic of cellular stress mechanisms. This may be linked to the enrichment of immune-related gene-sets like the innate immune response (GO:0045088), or biological processes such as T-cell receptor signalling pathways (GO:0050852). An EnrichmentMap showing upregulated gene-sets is displayed in Supplementary Figure S2.

ORA using *downregulated* genes revealed GO terms such as muscle contraction (GO:0006936) and synaptic activity (GO:0060025) consistent with the previously reported decrease in locomotion in this model [26]. During muscle contraction, the release of acetylcholine from motor neurons causes a depolarisation of the muscle membrane leading to an influx of calcium ions, initiating a further release of calcium ions from the sarcoplasmic reticulum. These calcium ions bind to the troponin-tropomyosin complex on the actin filaments and initiate the process of muscle contraction. Here, however, we note the downregulation of muscle contraction by calcium ion signalling (GO:0010882) involving the downregulation of genes such as *ERP44*, *PKD2*, *DMD*, and *CASQ2* which might impact intracellular calcium concentrations. Studies have shown that PKD2 binds with RyR2, a Ca^2+^ release ion channel on the sarcoplasmic reticulum, and inhibits Ca^2+^ release, with PKD2 knockout studies demonstrating increased Ca^2+^ oscillations and reduced levels of SR luminal Ca^2+^ [55]. Similarly, *CASQ2* encodes a protein that forms a complex with RyR2, were protein tetramers can bind up to 40 Ca^2+^, acting as a Ca^2+^ buffer that promotes reuptake into the sarcoplasmic reticulum [56]. Moreover, we note the downregulation of *DMD*, a gene encoding the dystrophin protein. Deficiencies in *DMD* are known to cause Duchenne muscular dystrophy, a disease found to be associated with increased intracellular Ca^2+^ concentrations [57], as the DMD protein acts as scaffolding for calcium ion channels and is therefore involved in regulating calcium ion homeostasis [58].

The malleability of the ECM means that it is sensitive to disuse [59], with hind-limb suspension models in rats showing decreases in collagens and increases in matrix metalloproteinases [59]. Unsurprisingly, we too have found a downregulation of ECM components such as fibronectin and collagen (GO:0062023) (Supplementary Figure S4), as well as a downregulation of focal adhesion (GO:0005925), basement-membrane, and cell junction (GO:0034330) GO terms involving *TNC*, *ITGA1*, *DAG1*, *COL6A6*, and *COL1A1* (Figure 5F). We also note an increase in *MMP2* expression, a matrix metalloproteinase involved in the degradation of collagens [60]. Moreover, changes to cytoskeleton genes were reportedly downregulated (GO:0030036, GO:0046785) with enrichment of the semaphorin-plexin signalling pathway (GO:0071526), which negatively regulates the cytoskeleton [61]. Additionally, several upstream regulators were predicted to be inhibited, such as *EZR*, involved in connecting the plasma membrane to the cytoskeleton, based on the downregulation of genes involved in cell motility (*ACTA1*) and focal adhesion (*PTK2*, *ITGA1*, *PXN*, *TLN1*) (Figure 5E). In addition, the protein digestion and absorption pathway were enriched, with *SLC9A3* and *ATP1A1* downregulated, as well as glycolysis-related processes (GO:0006090, GO:0006007, GO:0061621). We also note the downregulation of the androgen receptor signalling pathway (GO:0030521), regulation of the intracellular steroid hormone receptor signalling pathway (GO:0033143), and muscle cell differentiation (GO:0051146, GO:0042692, GO:0014902) (Figure 5B1). An EnrichmentMap showing downregulated gene-sets is displayed in Supplementary Figure S4.

### 3.3. Radiation Exposure under Induced Torpor Leads to Endoplasmic Reticulum Stress

**Figure 6:**
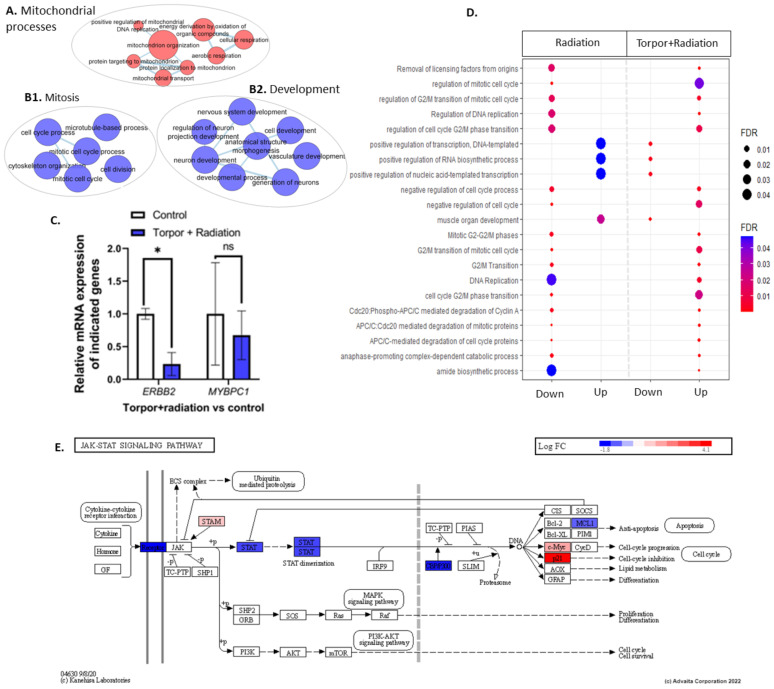
(**A**) shows an EnrichmentMap displaying upregulated GO terms related to mitochondrial processes. Clusters (**B1**,**B2**) show GO terms related to mitosis and development, respectively. (**C**) shows the qPCR analysis that details the significant downregulation of *ERBB2* and non-significant decrease in *MYBPC1*. (**D**) shows GO terms dysregulated between the radiation group (28.5-Crtl) and the induced torpor+radiation group (18.5-mel-rad). (**E**) shows the downregulation of genes involved in the JAK/STAT pathways. * *p* < 0.05, ns = non-significant.

To understand if the induced torpor model in the zebrafish protects against radiation in the muscle, DEA of the induced torpor+radiation versus the control group (18.5-mel-rad vs. 28.5-Ctrl) was performed, revealing upregulation of 628 DEGs and downregulation of 444 DEGs (*q* ≤ 0.1) (Table S10) that were used in ORA and impact analysis (Tables S11 and S12). Like that of the torpor-only group, ORA of the upregulated genes highlighted an increase in mitochondrial-based energy production including oxidative phosphorylation (GO:0006119, KEGG: 00190) (Supplementary Figure S5), the ETC (GO:0022900), with upregulation of *COXBA*, *COX7A2*, *NDUFA5*, and *NDUF96*, as well as mitochondrial translation GO terms (GO:0042775, GO:0019646, GO:0033108 1268842) (Figure 6A). Similarly, we observed changes that could impact proteostasis, such as the proteasome pathway and amino acid metabolism (GO:0006521), as well as ribosome biogenesis (GO:0042254, GO:0042273, GO:0042274), and amino acid biosynthesis (KEGG: 01230) with upregulation of *CBS* and *GLUL*. Moreover, metabolic genes were also enriched for both carbohydrates (GO:1901135), and ketones (GO:0042180). Like the radiation group, we observed enrichment of the unfolded protein response (GO:0006986, GO:0035967) and response to endoplasmic reticulum stress (GO:0034976), which might suggest radiation-induced protein damage. Again, we observed a response to hypoxia and low oxygen levels (GO:0001666, GO:0036294), as well as a response to oxidative stress and superoxide (GO:0006979, GO:0000303).

Interestingly, ORA of *downregulated* genes included the cytokine-cytokine receptor interaction pathway with receptor genes for IL2, IL6/12, TNF, and TGF-β, as seen in Figure S7. In contrast with the radiation group, the JAK-STAT signalling pathway (KEGG: 04630) was downregulated involving *STAT6* and *EP300* (Figure 6E). In addition, we observed downregulation of terms related to muscle cell differentiation, development, and morphogenesis (GO:0042692, GO:0014706), with *ERBB2*, *MYOT*, and *MYBPC1* downregulated along with development, and morphogenesis of vasculature (GO:0001944, GO:0048514) (Figure 6B2). qPCR analysis of *ERBB2* showed a significant downregulation (*p* = 0.03), while downregulation of *MYBPC1* (*p* = 0.6) was non-significant (Figure 6C). Moreover, cytoskeleton binding and organisation was enriched as well as vinculin binding (GO:0017166), focal adhesion (GO:0005925), cell junction organization (GO:0034330), and cytoskeleton organisation (GO:0007010 GO:0030036). This coincided with the downregulation of the cytoskeleton-related genes *DMD*, and *SYMN,* which function in connecting the cytoskeleton to the ECM. EnrichmentMaps were generated using up and downregulated genes and are presented in Supplementary Figures S7 and S8.

We next compared the induced torpor+radiation group to both the torpor group and the radiation group, to characterise the response. The comparison between the induced torpor+radation group and the radiation group (18.5-mel-rad vs. 28.5-rad *q* ≤ 0.4) revealed 25 common upregulated genes, and 51 common downregulated genes (Table S13). ORA of the *upregulated* genes suggests shared aminopeptidase activity (GO:0004177), muscle development and differentiation (GO:0035914, GO:0007517), translation (GO:0006418), and an unfolded protein response (1268756) (Table S14). Several of the over-represented terms using the shared *downregulated* genes revealed cell cycle and division (GO:0044772, GO:0000280, GO:0140014), reproductive processes (GO:2000241, GO:1905881), and translation (GO:0006417). Additionally, a comparison was made with GO terms generated from the radiation and torpor+radiation groups. Shared GO terms regulated in the same direction are displayed in Figure S9, showing shared upregulation of oxoacid/organic/carboxylic acid metabolism and a shared downregulation of GO terms related to protein kinase activity, organelle fission, cytoskeleton organisation, and cyclin-CDK complex. In addition, eight genes were upregulated in the radiation group and downregulated in the induced torpor+radiation group including *FBXO32*. *FBXO32* is highly expressed during muscle atrophy [62]. While four genes were downregulated in the radiation group and upregulated in the induced torpor+radiation group including *MAP2K6*. It plays a role in signal transduction and the stress response. GO terms regulated in opposite directions are presented in Figure 6D, showing the upregulation of mitotic GO terms in the torpor+radiation group that were downregulated in the radiation group while developmental and transcription related terms were upregulated in the radiation group and downregulated in the torpor+radiation group.

The comparison between the induced torpor+radiation group and the induced torpor group (18.5-mel-rad vs. 18.5-mel, *q* ≤ 0.1) revealed 367 shared upregulated genes and 185 shared downregulated genes (Table S15). ORA of the shared *upregulated* genes revealed energy production such as oxidative phosphorylation and the ETC (GO:0006119, GO:0022900) in addition to mitochondrial translation (1268843) and gene expression (GO:0140053), ribsome biogenesis and translation (GO:0042254, GO:0006412), protein folding (GO:0006457), and the proteasome pathway (83040). ORA also indicated response to hypoxia (GO:0071456), oxidative stress (GO:0006979) and cell cycle progression (1269831, 1269799), and p53-Dependant DNA damage checkpoints (1269744) (Table S16).

### 3.4. Induced Torpor Led to Significantly Less DNA Damage than Low Dose Radiation

**Figure 7:**
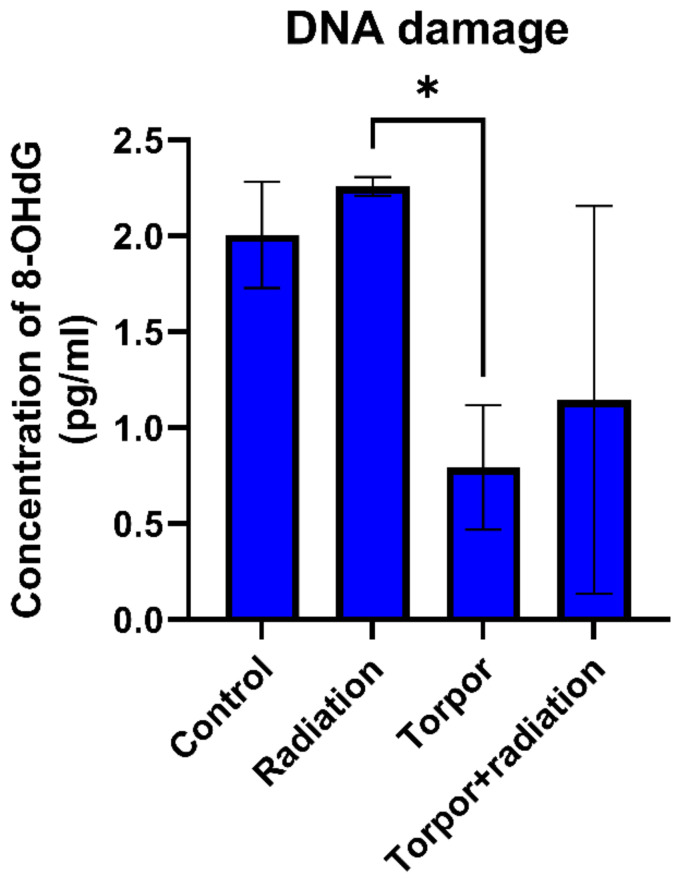
DNA damage for each experimental group. Levels of DNA damage were significantly lower in the torpor group versus the radiation group. * *p* < 0.05.

Results from the DNA damage assay showed that radiation led to a non-significant (*p* = 0.165) increase in DNA damage (8-OHdG levels) in comparison to the control group. As seen in Figure 7, we also observed a significant decrease in DNA damage when comparing the torpor group and the radiation group (*p* = 0.0122). A decrease in DNA damage was also shown when comparing the torpor+radiation group and the radiation group, with results approaching significance (*p* = 0.0569).

### 3.5. Shared Responses to Hypothermia and Inactivity in Hypothermic Zebrafish and Hibernating Brown Bear

**Figure 8:**
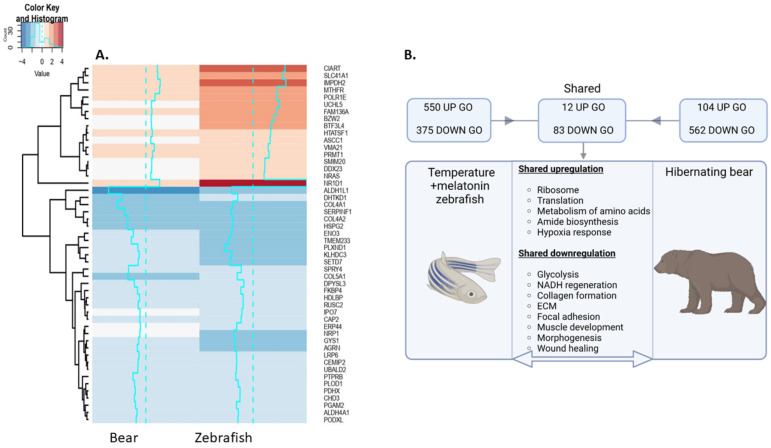
(**A**) Heatmap depicting up and downregulated genes shared between the torpor group zebrafish model and hibernating bear gastrocnemius muscle. (**B**) Summary of shared GO terms in these groups. Notably, we find shared upregulation of genes involved in translation and amino acid metabolism, as well as shared downregulation of ECM and developmental genes. Created with BioRender.com.

We wanted to determine whether similarities exist in the transcriptomic response of the hypothermic state zebrafish muscle with that of the gastrocnemius muscle of hibernating bear, which was chosen for a fast twitch muscle comparison. Transcriptomic analysis of the hibernating bear muscle vs active control revealed 434 upregulated DEGs and 569 downregulated DEGs (FC ± 1.5, *q* ≤ 0.1) (Table S17) which were subject to Toppfun enrichment and impact analyses using iPathwayGuide (Tables S18 and S19). A comparison of the DEGs in the zebrafish torpor group and the hibernating bear revealed 89 shared genes (*q* ≤ 0.1) (Table S20), of which 17 were upregulated and 33 were downregulated, as shown in Figure 8A. A comparison of up and downregulated GO terms is found in Table S21, along with ORA of genes expressed in opposite directions. The comparison of GO terms generated from each dataset was made as summarised in Figure 8B. This revealed shared upregulated GO terms related to the ribosome (GO:0005840), translation (GO:0006412), response to hypoxia (GO:0001666), and metabolism of amino acids (1270158). Shared downregulated GO terms included ECM (GO:0031012, M7098), focal adhesion (M7253), basement membrane organisation (GO:0071711), and collagen biosynthesis and modification (GO:0005581, 1270246). Several gene-sets related to muscle and tissue development/morphogenesis (GO:0007517, GO:000156, GO:0009887, GO:0007517, GO:0060537), glycolysis (PW:0000025, GO:0006007, GO:0006096), and NADH regeneration (GO:0006735) were also enriched. ORA of genes differentially expressed in opposite directions suggests the torpor group in zebrafish had more upregulated genes involved in amino acid metabolism (SMP00032), ETC (1270128), and oxidative phosphorylation (PW:0000034). To explore the link between downregulation of ECM and development, we performed a network analysis with GO terms, extracellular matrix organisation, and muscle organ development in both the zebrafish torpor group and the hibernating bear muscle. In Figure S10A we show an interaction between *COL6A3* and *DAG1* with the muscle organ development GO term in the zebrafish while Figure S10B shows an interaction between *LAMA2* and the muscle organ development GO term.

## 4. Discussion

### 4.1. STAT3-Mediated Regeneration Events in the Muscle following Low Dose Radiation

Muscle injury can result in necrotic muscle cells where damaged cell membranes release cellular contents and chemotactic factors that invoke a repair event for muscle regeneration. In the early stages of muscle regeneration, these chemotactic molecules attract immune cells such as neutrophils, mast cells, and macrophages that help clear cell contents and damaged muscle cells [63]. During the repair phase, myofibres, macrophages, and T cells produce IL-6 [64] which promotes proliferation and differentiation of myoblasts through the STAT3 signalling pathway [65]. Studies have shown the importance of STAT3 after injury, as knockout of *STAT3* in muscle stem cells shows impaired regeneration [66,67]. The binding of IL-6 to its receptor (IL-6R) leads to the formation of a heterohexameric complex that activates the JAK/STAT3 pathway where activation of STAT3 leads to its translocation to the nucleus to initiate transcription of its target genes [68]. When IL-6 binds to its receptors on muscle stem cells (satellite cells) and myoblasts (muscle progenitor cells), it initiates transcription of *MYOD1* which itself initiates transcription of muscle-specific genes such as myosin heavy and light chains, to promote their differentiation to myotubes. These cells eventually mature into myofibres that replace the necrotic cells [69].

The radiation group shows enrichment in IL-6 signalling pathways and cell differentiation GO terms with the upregulation of *STAT3* and *MYOD1*; accordingly, repair events might be taking place as part of muscle regeneration. Additionally, our results are in line with spaceflight studies reporting enrichment of both IL-6 and STAT signalling in the fast-twitch EDL of space-flown mice [70,71]. There is much to be learnt about IL-6 secretion in muscle in response to LDR exposure, however, it is secreted by endothelial cells in response to radiation [72]. While IL-6 and STAT3 mediate regeneration through the activation of myocytes and satellite cells, chronically elevated levels have been implicated in skeletal muscle atrophy [73,74]. In summary, our results suggest that the upregulation of *STAT3* and its downstream effector, *MOYD1*, could signal cytokine-mediated muscle cell regeneration in response to insult. Furthermore, STAT3’s anti-proliferative characteristics make it a compelling target for further research exploring the impact of chronic radiation exposure on muscle mass during spaceflight.

### 4.2. Shared Mechanisms of Atrophy Resistance

As noted above, hibernating animals have evolved the unique ability to resist muscle atrophy in the face of catabolic stimuli such as inactivity and fasting which would otherwise lead to the activation of autophagy or proteasomal degradation and result in muscle loss. One study exploring the mechanisms of atrophy resistance in the muscle of hibernating bear focused on the balance between pro-atrophy TGF-β and anti-atrophy BMP pathways and found that during hibernation pro-atrophy TGF-β-related genes were downregulated while BMP-related genes were upregulated. TGF-β facilitates muscle atrophy through SMAD2/3 signalling by upregulating E3 ubiquitin ligases, such as TRIM63 and FBXO32 [75,76]. For instance, FBXO32 targets c-Myc for degradation, which is involved in cell growth [62]. Hence, *FBXO32* upregulation has been observed during muscle atrophy [62]. Interestingly, we observed an increase in expression of *FBXO32* (log2FC = 1.42, *q* = 1.4) in the radiation group, pointing to a mechanism by which low-dose radiation exposure during space flight might contribute to muscle loss. In contrast, this gene was observed to be downregulated in the induced torpor+radiation group, suggesting a potential induced torpor-like state-related protective effect against muscle loss. When looking to the literature, we note that both melatonin treatment and hypothermic conditions can lead to a decrease in *FBXO32* (*MAFbx*) to ameliorate muscle atrophy [77,78]. In the hibernating bear muscle, *FBXO32* showed a non-significant decrease but a significant upregulation of *MYC*. In addition, *FBXO32* is downregulated in the muscle of hibernating arctic ground squirrel [79]. We also report a shared increase in ribosome biogenesis and translation, and a reduction in protein catabolism in the zebrafish torpor group with the muscle of hibernating bears, which has been hypothesised to contribute to atrophy resistance during the inactivity of torpor [80]. However, we note that functional tests were not performed to assay muscle size and thus further work is needed to determine whether muscle atrophy is ameliorated during inactivity in the hypothermic zebrafish, and the roles of *FBXO32* and *MYC* in mediating this.

### 4.3. The Link between the Extracellular Matrix and Development

The ECM of the skeletal muscle is made up of collagens, glycoproteins, proteogylcans, and elastins with structural and functional properties [81] and a role in the growth [82], repair [83], and transmission of contractile forces [84]. Here, we describe a decrease in expression of genes involved in the ECM in the induced torpor and induced torpor+radiation groups, a phenotype also observed in the hibernating bear. The ECM is a highly dynamic connective tissue that changes in response to use. On one hand, a stimulus such as exercise increases ECM components at both the mRNA and protein levels [85], while factors such as age and disuse contribute to a decline in ECM integrity, as demonstrated in comparisons of the ECMs in soleus muscles of young and old mice [86]. Similarly, in a model of inactivity, hind-limb unloading studies found a decrease in expression of ECM-related genes such as type 1 collagen and laminin in the soleus [87]. Hence, this downregulation of ECM genes in the zebrafish and hibernating bear is consistent with the reduction seen during disuse or aging and is likely to be directly linked to inactivity. However, exogenous administration of exogenous melatonin has also been shown to downregulate components of the ECM such as collagen 1 and fibronectin, and, as such, to prevent ischemia-related fibrosis in murine muscle [88]. Moreover, in a similar study exploring the effects of hypothermia on connective tissue in the compact myocardium, zebrafish were acclimated to 20 °C from 27 °C, resulting in a decrease in collagen content and increase in expression of matrix metalloproteinase genes at the reduced temperatures [89].

A striking association emerged in the data involving the downregulation of ECM presenting along-side the downregulation of development-related genes in both the hypothermic zebrafish groups and the hibernating bear. This is of interest given the multi-faceted role of the ECM in cell and tissue development via processes such as migration, morphology, cell adhesion, polarity, and differentiation, all of which are integral in orchestrating successful tissue development [90]. We know that the ECM plays an important role in the repair response for example, as connective tissue such as fibronectin and collagen are produced by fibroblasts and activated MSCs to form granulation tissue that provides a dermal matrix framework for cell migration to form new tissue [91]. The relationship between ECM components and development has been made clear in studies showing that the inhibition of collagen synthesis inhibits myoblast differentiation [92]. Moreover, the role of integrins in transducing developmental signalling is evident from studies showing that the blocking of the integrin receptor complex also prevented myoblast differentiation and myotube fusion [93], which are important for muscle regeneration. Here, we performed a network analysis to explore the relationships between pathways and gene-sets of downregulated ECM and developmental genes that highlighted *COL6A3* and *DAG1* as key regulators of muscle organ development gene-set in the zebrafish, and *LAMA2* in the bear. Mutations of these genes contribute to a muscular dystrophy phenotype [94,95,96] characterised by muscle wasting and muscle weakness. In addition, studies have found that dystroglycan (*DAG1)*, a protein that links the ECM and cytoskeleton in skeletal muscle, has a role both in embryonic development [97] and muscle regeneration [98], further demonstrating the link between the ECM components and the regulation of development.

In a study exploring the response to cardiotoxin-induced muscle injury in the skeletal muscles of hibernating squirrels, Andres-Mateos et al. [99] observed inflammation, macrophage infiltration, and degenerating fibres. However, they found an absence of developmental markers (myosin) or morphological changes 4 days’ post-injury, with the first regenerating fibres appearing 6-weeks post-injury, indicating a delay in muscle regeneration during hibernation. Andres-Mateos et al. found significantly less fibrotic tissue accumulation 6 weeks’ post-injury in comparison to controls, suggesting decreased ECM deposition, and at 4 weeks after arousal from torpor, the muscle made a complete recovery without the presence of fibrotic tissue. They also found decreased levels of TGF-β1, TNF-α, and IL-6 during torpor that they suggest might prevent fibrogenesis, despite the presence of inflammation. In comparison, our results for the torpor+radiation group show a downregulation of the cytokine-cytokine receptor interaction pathway involving receptor genes for IL2, IL6/12, TNF, and TGF-β. This, along with the downregulation of JAK/STAT signalling, and development/differentiation genes, that are upregulated in the radiation group, is consistent with delayed regeneration. We also note that previous studies show that melatonin can enhance muscle regeneration after injury by promoting expression of differentiation markers, however, the downregulation of developmental GO terms might suggest that hypothermia is exerting the dominant effects on the muscle cells [100].

Few studies have explored the effects of cold temperature exposure upon ECM dynamics. One study focusing on cold acclimated zebrafish found a decrease in collagen content in the pericardial membrane of a zebrafish heart with an increase in expression of matrix metalloproteinases [89]. On the other hand, a relationship between reduced temperatures and reduced developmental rates has been demonstrated consistently throughout the literature [101], with one such study showing decreased developmental markers (somites) in zebrafish maintained at lower temperatures. They also showed that these zebrafish were larger than those maintained at increased temperatures when developmental stages were corrected for, which has been associated with a better physical condition [102] and suggests that growth can occur independently of development [103]. To concur, studies have also revealed that ectotherms inhabiting low temperatures experience an increase in lifespan [104], a phenomenon that has been observed in humans, where a lower body temperature is an indicator of longevity [105]. The use of ectothermic animals may therefore prove useful in defining the relationship between ECM regulation, development, and growth and further work may shine light on therapeutic pathways for developmental myopathies. To continue, there are limited studies observing the effects of both melatonin treatment and hypothermic conditions.

### 4.4. Dysregulated DNA Repair Pathways in the Muscle Post-Irradiation

In both the radiation group and the induced torpor+radiation group, we report the enrichment of several cell cycle/division-related gene-sets using downregulated genes that might suggest some cells were undergoing radiation-induced cell cycle arrest or cellular senescence [106,107]. Additionally, while it is well established that DNA repair mechanisms are activated following radiation exposure, our results suggest a downregulation of DNA repair pathways 2 days’ post-irradiation involving genes such as *RAD51C*, which functions in homologous recombination. In a similar study of low dose radiation exposure, Suman et al. [108] performed DNA damage assays on mice intestines 2 months post-irradiation (0.5 Gy) using gamma and iron radiation. Like the results reported here, low dose gamma radiation did not lead to a significant increase in DNA damage compared with controls, however, they did report that it led to decreased expression of DNA repair pathways, as reported here. Therefore, damage accrued from LDR may occur over time due to the diminished ability of a cell to repair DNA. We previously reported that the induced torpor model led to the upregulation of DNA repair in the gastrointestinal tract and liver, which was sustained even with radiation exposure, however, DNA repair gene-sets were not enriched in the muscle, suggesting a tissue-specific response. In addition, hypothermic states can lead to ROS generation that can damage DNA, suggested by the upregulation of genes involved in p53-mediated DNA damage checkpoints in the torpor group, however, the results show less DNA damage than that of the radiation group. On the other hand, a non-significant decrease was observed in the torpor+radiation vs. radiation comparison. Given that previous work has shown that hypothermic states confer radio-protection [109,110,111], our results may be limited by sample size. It has been postulated that one of the mechanisms of the protection afforded by a hypothermic/hypometabolic state is due to the ‘*oxygen effect*’, where hypoxic environments restrict ROS generation. Granted, the addition of melatonin, which has been shown to possess antioxidant properties, may also be contributing to a reduction in DNA damage. Alternatively, a reduction in movement or reduction in metabolism in comparison to controls might also result in reduced ROS generation. Further work is required to dissect the effects of temperature and melatonin in reducing DNA damage in this model, with larger sample sizes. Finally, the idea that LDR can compromise DNA repair pathways in muscle post-irradiation, leading to the accumulation of DNA damage over time, points to the need for ongoing treatment post-space-travel/irradiation. Future research directions will seek to validate whether long-term deficits in DNA repair capacity persist and whether supplementation with antioxidants can mitigate accumulative damage.

### 4.5. Increased Mitochondrial Transcriptional Activity in Muscle as a Cold Adaptive Strategy to Maintain Energy Homeostasis

We postulate that increased mitochondrial transcriptional activity in the muscle of cold-tolerant species is a cold adaptive strategy to enable favourable energy homeostasis that may contribute to the maintenance of muscle mass. In humans, periods of chronic inactivity disrupt mitochondrial dynamics leading to changes in energy homeostasis that can in turn produce muscle atrophy. These effects are seen during aging, malnutrition, or in genetic conditions such as mitochondrial myopathy where mitochondrial dysfunction is present. During disuse, for example, muscle cells adapt by decreasing mitochondrial biogenesis and increasing mitophagy, resulting in a diminished mitochondrial network [112,113]. Similarly, disuse leads to a downregulation of genes involved in oxidative phosphorylation, decreasing the efficiency of mitochondrial respiration [114]. Inactivity also reduces antioxidant capability in muscle cells, leading to increased ROS that advances atrophy by repressing protein synthesis, and stimulating both proteolytic degradation and apoptotic pathways [115,116]. Mitochondrial dysfunction is prominent in patients with myopathy who exhibit genetic mutations in mitochondrial DNA or a reduced copy number of genes involved in oxidative phosphorylation causing muscle atrophy and weakness. It is characterised by reductions in mitochondrial quantity and quality with fibre type shifting towards a glycolytic phenotype [117]. Similarly, sarcopenia, which relates to muscle loss due to aging, is characterised by a loss of strength, decreased metabolic rate, and diminished aerobic capacity [118]. Muscle atrophy also occurs in microgravity, and spaceflight studies consisting of multi-tissue analysis of space-flown animals confirmed mitochondrial dysfunction as a systemic stress response, including in the muscles [119], with further models of hypogravity showing that muscles undergo a slow-to-fast twitch conversion, similar to that seen in mitochondrial myopathy [120]. It is known that fast twitch muscles are more susceptible to atrophic conditions than slow twitch muscles, particularly through the FOXO family, the TGFβ family, NF-κB, and disuse-related pathways [121]. In addition, studies in sarcopenia demonstrate oxidative muscles such as the soleus are more resistant to age-related atrophy [118]. Conversely, spaceflight studies have revealed the slow twitch soleus to be particularly atrophy-prone [18], which may therefore be due to the observed slow-to-fast twitch fibre conversion that might compromise energy homeostasis [122].

Interestingly, our results show that the cold-tolerant zebrafish experiencing hypothermic conditions in this study had elevated expression of genes involved in mitochondrial transcription and translation in the dorsal muscle, indicative of an oxidative phenotype with more capacity for generating ATP. This has also been reported in other ectotherms such as eels, striped bass, carp, and goldfish that experience fluctuations in environmental temperatures. As temperature can affect biochemical reactions, the ability to generate ATP through an increase in oxidative enzymes is a compensatory method in which these animals counteract temperature-induced reductions in metabolic rate [123]. They have therefore been found to contain larger mitochondria due to increased protein turnover, which may increase mitochondrial efficiency [124]. A study examining the effects of cold acclimatisation (from 28°C to 18°C) and exercise in zebrafish found that both cold exposure and exercise independently increased mitochondrial genes such as citrate synthase, β-, and Cytochrome c oxidase, with both conditions experiencing an upregulation of NRF-1, a downstream effector of PGC1α: a master regulator of mitochondrial biogenesis [125]. This shows that cold exposure and exercise increase mitochondrial biogenesis through a common mechanism. We also report increases in expression of *TFAM* whose transcription is activated in a NRF1-dependent manner. Likewise, the addition of melatonin may be having additive effects on mitochondrial dynamics as it has also been shown to increase mitochondrial biogenesis through the PGC1α signalling pathway [126]. Similarly, studies in cold-acclimatised Drosophila have also shown higher mitochondrial coupling and ATP synthesis and presented with increased survival compared with controls after cold exposure, suggesting a conserved response to cold exposure in cold-tolerant species [127]. Furthermore, we previously described a decrease in genes involved in calcium uptake in the sarcoplasmic reticulum, which might suggest greater cytosolic Ca^2+^ concentrations. Previous studies have shown that increases in intracellular Ca^2+^ concentrations can occur in response to hypoxic conditions [128]. This is important given the role of Ca^2+^ in generating muscle contractions, and its role in stimulating ATP production by modulating enzymes involved in the TCA cycle. However, studies in neurons have demonstrated that hypothermia has a neuroprotective effect on hypoxia-related increases in intracellular Ca^2+^ concentrations, and so further study is required to examine the Ca^2+^ dynamics [129]. Furthermore while mitochondria have been known to act as Ca^2+^ buffers, sharp increases in intracellular Ca^2+^ concentrations can overload mitochondria, leading to the activation of the mitochondrial permeability transition pore that can initiate the apoptotic process [130]. Conversely, hypothermic conditions have been found to increase mitochondrial calcium loading capacity and lower the activation of the mitochondrial permeability transition pore [131]. Hence, if the observed increase in genes involved in mitochondrial transcription and translation, translate to an increase in mitochondrial capacity or biogenesis, then mitochondria may be playing a greater role in buffering a spike in intracellular Ca^2+^ concentrations in the muscle to maintain calcium homeostasis.

In comparison to humans, hibernating animals possess a unique ability to limit muscle atrophy during disuse. While our results for the hibernating bear muscle show a downregulation of mitochondrial genes during hibernation, studies have shown that an increase in genes involved in mitochondrial biogenesis occurs post-hibernation in bears [20]. The maintenance of muscle mass in bears may therefore be more dependent on their ability to maintain nitrogen balance in the muscle, which prevents protein utilisation for energy during hibernation [132]. On the other hand, the literature from the 13-lined ground squirrels, which do not recycle nitrogen from urea during torpor, shows an increase in mitochondrial biogenesis in the muscle with a fast-to-slow twitch conversion, in a process mediated by PCG-1α [133]. This process can be likened to that which occurs in humans after exercise. Additionally, this phenotype is in direct contrast to the slow-to-fast phenotype conversion seen during spaceflight [120,134]. Similarly, the mitochondrial number was found to increase during torpor in both fast and slow twitch muscles (extensor digitorum longus and soleus) of the Daurian ground squirrels (*Spermophilus dauricus*), pointing to increased mitochondrial capacity [135], and a study of mitochondrial respiration in hibernating ground squirrels found that mitochondrial respiration did not decrease during torpor [136]. Given that hibernating animals resist disuse-related atrophy during torpor, and slow twitch muscles are more atrophy resistant, the maintenance or establishment of a slow oxidative phenotype in some hibernating animals may represent a key mechanism behind muscle mass conservation, and highlights its potential for therapeutic intervention to avoid muscle atrophy during spaceflight. The literature, therefore, supports a theory of increased mitochondrial biogenesis/capacity and phenotype shifting as a conserved response among some ectothermic and heterothermic animals in cold adaptation. Some work has been carried out to determine whether genes related to mitochondrial biogenesis are activated in humans upon cold exposure, however, the results suggest that exercise is needed alongside cold exposure to elicit such a response [137,138]. It has previously been shown that low-frequency electrostimulation can induce a fast-to-slow fibre conversion in paraplegic patients [139]. Hence, in the context of induced torpor in humans, it must be asked whether hypothermia coupled with electrostimulation might encourage mitochondrial biogenesis as a means of maintaining a slow oxidative phenotype and conferring resistance to atrophy. Alternatively, the administration of pharmaceutical agents to increase mitochondrial biogenesis might work to combat muscle atrophy during disuse.

## 5. Conclusions

In summary, we found that LDR in the muscle of the zebrafish led to an inflammatory immune response with enrichment of NF-kβ and IL-6 signalling and upregulation of the STAT3 transcription factor. In contrast, results for the torpor+radiation group showed the downregulation of the cytokine-cytokine receptor pathways involving IL2, IL6/12, TNF, and TGF-β receptors, as well as a downregulation of the JAK/STAT pathway, consistent with a torpor induced suppression of the immune system seen in hibernating animals. In the radiation group, we also observed an upregulation of genes involved in differentiation and regeneration featuring the upregulation of *MYOD1* suggesting muscle remodelling. Conversely, developmental genes and pathways were downregulated in the torpor groups. Moreover, we observed the downregulation of ECM genes in the induced torpor groups, with or without radiation, consistent with a decrease in physical activity, and explored the link between the downregulation of ECM and developmental genes highlighting *COL6A3* and *DAG1* as linking factors. We also highlight the differential regulation of *FBXO32* between the radiation group and the induced torpor groups, which might have implications in torpor-related protection against muscle atrophy. Interestingly, we observed an increase in genes related to mitochondrial biogenesis in the zebrafish torpor groups that have been observed during torpor in hibernating animals, which might confer atrophy resistance by conferring or maintaining a slow oxidative phenotype in muscles. Overall, our results provide novel insights into how low-dose radiation might impact muscle biology in the context of spaceflight. In addition, the induced torpor model shows utility in modelling specific processes also seen in the muscle of hibernating animals and the analysis of the torpor+radiation group revealed the presence of markers that might confer radioprotective benefits, and warrant further investigation.

## Figures and Tables

**Table 1:** Experimental groups showing the group names, keys, sample numbers per condition, values of radiation exposure, ambient water temperatures, and melatonin treatments.

Group	Key	Sample (N)	Radiation (cGy)	Water Temperature (°C)	Melatonin (µM)
Control	28.5-Ctrl	6	0	28.5	0
Radiation	28.5-rad	6	32.64	28.5	0
Temperature+melatonin (Induced torpor)	18.5-mel	6	0	18.5	24
Temperature+melatonin+radiation (Induced torpor+radiation)	18.5-mel-rad	6	32.64	18.5	24

## Data Availability

The data that support the findings of this study have been submitted to the NCBI Gene Expression Omnibus, and can be found at: https://www.ncbi.nlm.nih.gov/geo/query/acc.cgi?acc=GSE222614 (accessed on 5 May 2023).

## Supplementary Materials

The following supporting information can be downloaded at: https://www.mdpi.com/article/10.3390/cells12101366/s1, Figure S1: Enrichmentmap of up and downregulated genes radiation vs control; Figure S2: Enrichmentmap of upregulated genes torpor vs control; Figure S3: IPG ECM receptor interaction for torpor vs control; Figure S4: Enrichmentmap of downregulated genes torpor vs control; Figure S5: IPG Oxidative phosphorylation torpor radiation vs control; Figure S6: IPG Cytokine cytokine receptor interaction; Figure S7: Enrichmentmap of upregulated genes torpor+radiation vs control; Figure S8: Enrichmentmap of downregulated genes torpor+radiation vs control; Figure S9: Bubble plot showing shared GO terms in torpor radiation vs radiation group; Figure S10: ECM development network analysis; Tables S1–S3: Reverse transcriptase qPCR protocol and primers description; Table S4: DEG Radiation vs control zeb muscle; Table S5: ORA Radiation vs control zeb muscle; Table S6: IPG radiation vs control zeb muscle; Table S7: DEG Torpor vs control zeb muscle; Table S8: ORA Torpor vs control zeb muscle; Table S9: IPG Torpor vs contorl zeb muscle; Table S10: DEG Torpor Radiation vs control zeb muscle; Table S11: ORA Torpor Radaition vs-control muscle; Table S12: IPG Torpor radiation vs control zeb muscle; Table S13: Shared genes in torpor radiation vs radiation; Table S14: ORA of shared genes in torpor radiation vs radiation; Table S15: Shared genes in torpor radiation vs torpor group; Table S16: ORA of shared genes in torpor radiation vs torpor group; Table S17: DEG urus arctos gastroc hib v act; Table S18: ORA hibernating vs active bear gastroc; Table S19: IPG Hibernating vs active bear gastroc; Table S20: DEGs shared in torpor vs control zebrafish vs hibernating vs active bear; Table S21: Comparison of GO in torpor zebrafish and hibernating bear and ORA of shared genes.
