# Supplementary figures and images for: Transcriptome Profiling Reveals Enhanced Mitochondrial Activity as a Cold Adaptive Strategy to Hypothermia in Zebrafish Muscle
Source: Cells. 2023 May 11;12(10):1366. doi: 10.3390/cells12101366 (PMC10216211; doi:10.3390/cells12101366)

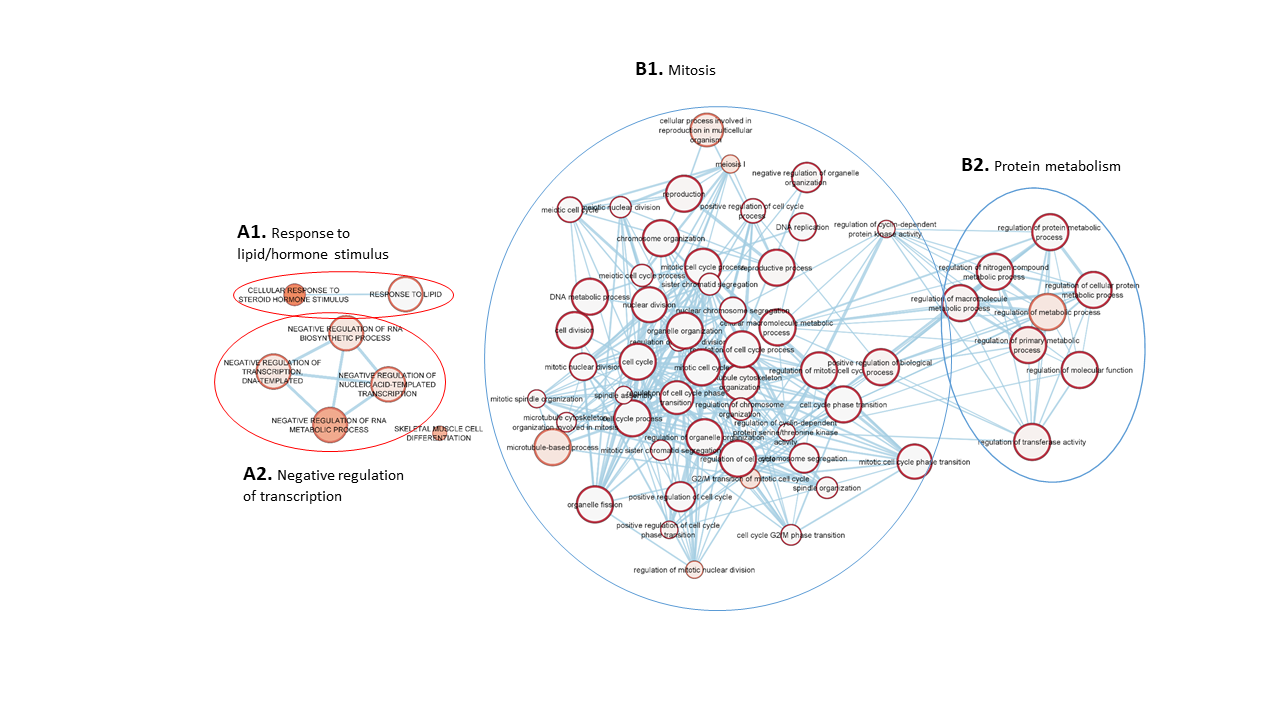

Supplement: Supplementary file 1 [file cells-12-01366-s001.zip › Supplementary_Figure_S1_Enrichmentmap_of_up_and_downregulated_genes_radiation_vs_control.tif]

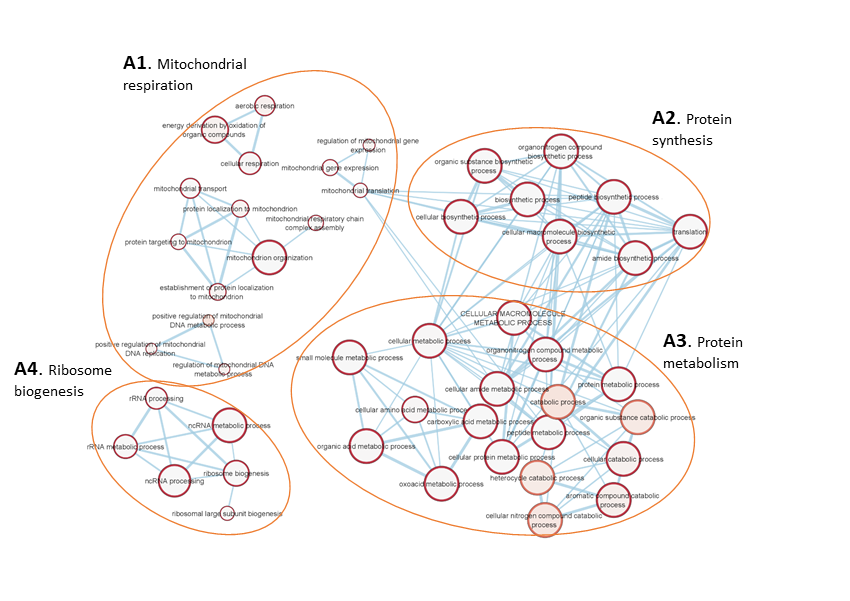

Supplement: Supplementary file 1 [file cells-12-01366-s001.zip › Supplementary_Figure_S2_Enrichmentmap_of_upregulated_genes_torpor_vs_control.tif]

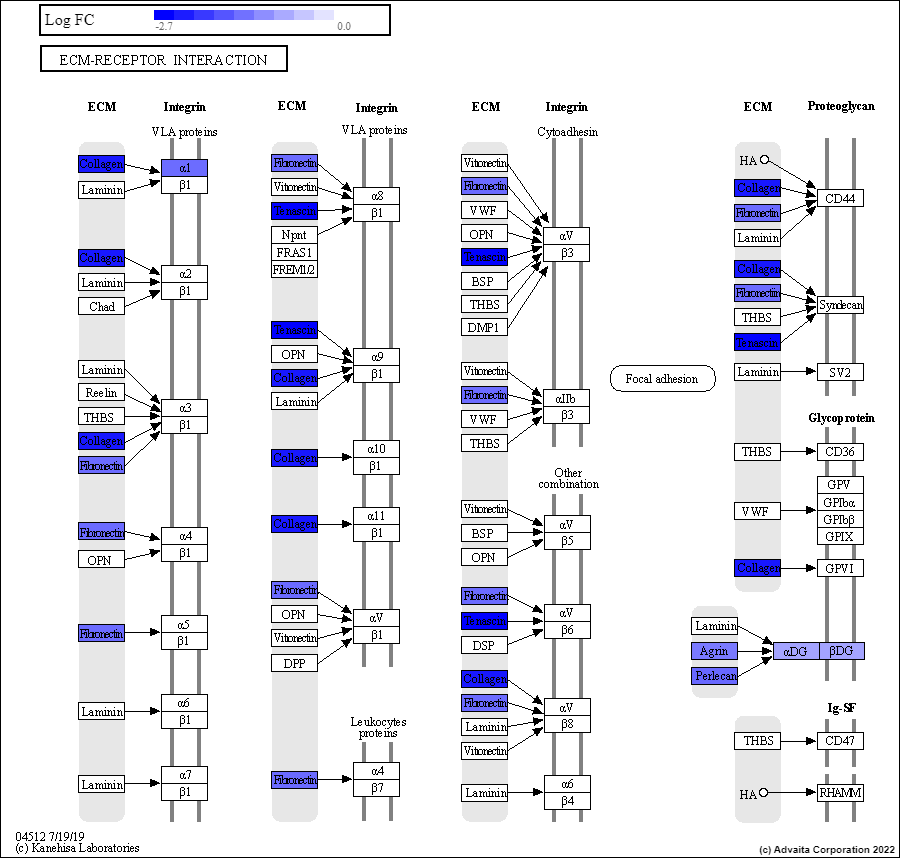

Supplement: Supplementary file 1 [file cells-12-01366-s001.zip › Supplementary_Figure_S3_IPG_ECM_receptor_interaction_for_torpor_vs_control.png]

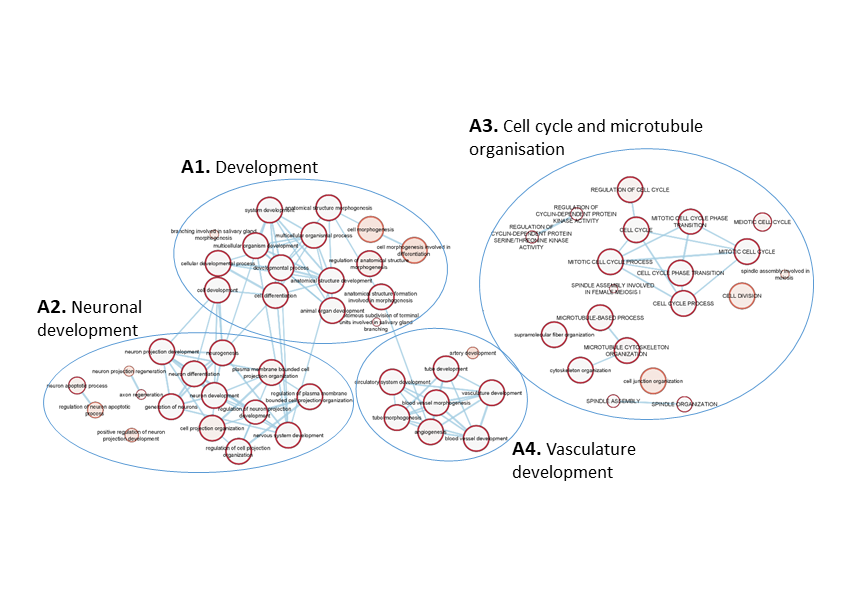

Supplement: Supplementary file 1 [file cells-12-01366-s001.zip › Supplementary_Figure_S4_Enrichmentmap_of_downregulated_genes_torpor_vs_control.tif]

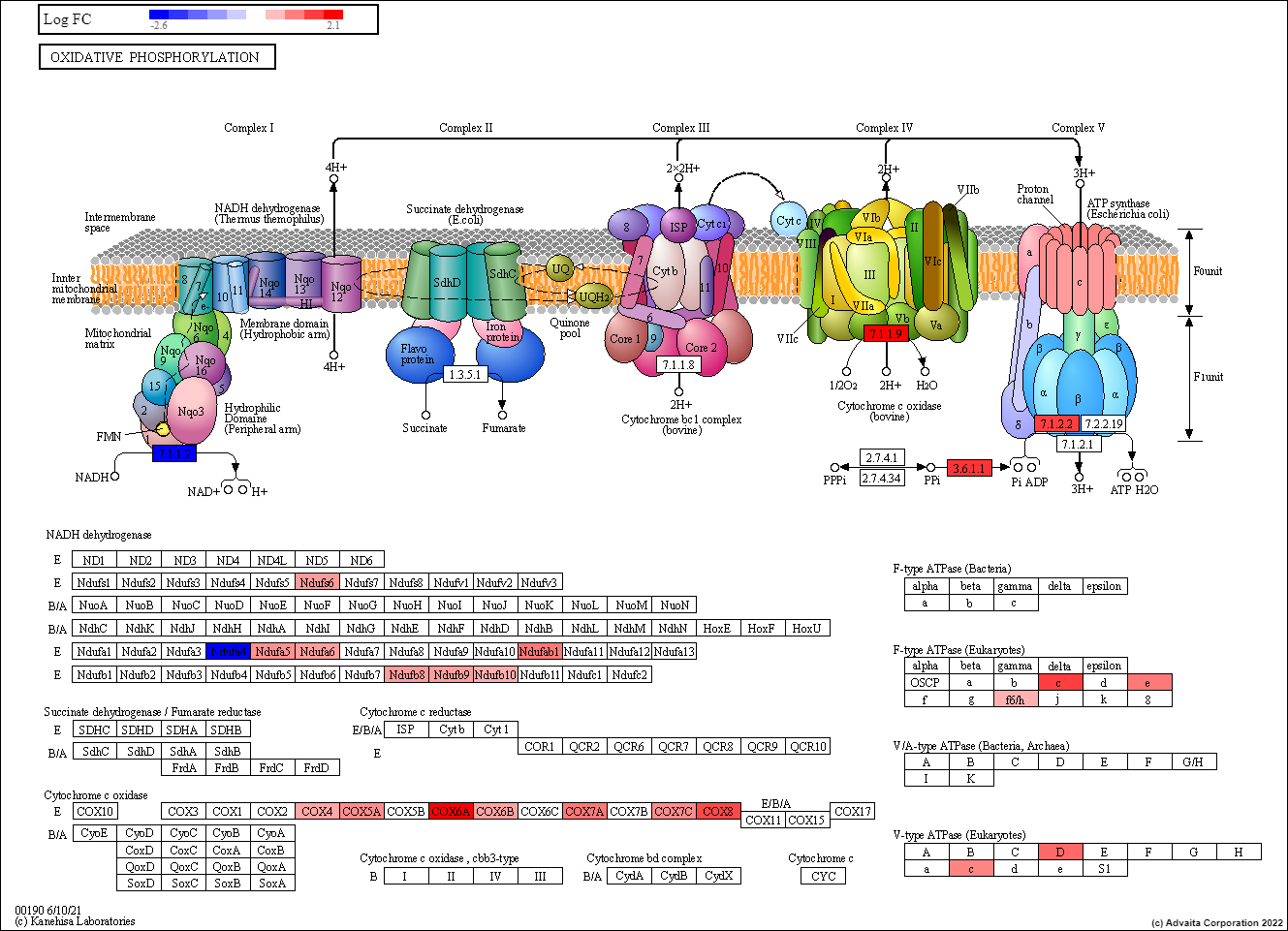

Supplement: Supplementary file 1 [file cells-12-01366-s001.zip › Supplementary_Figure_S5_IPG_Oxidative_phosphorylation_torpor_radiation_vs_control.png]

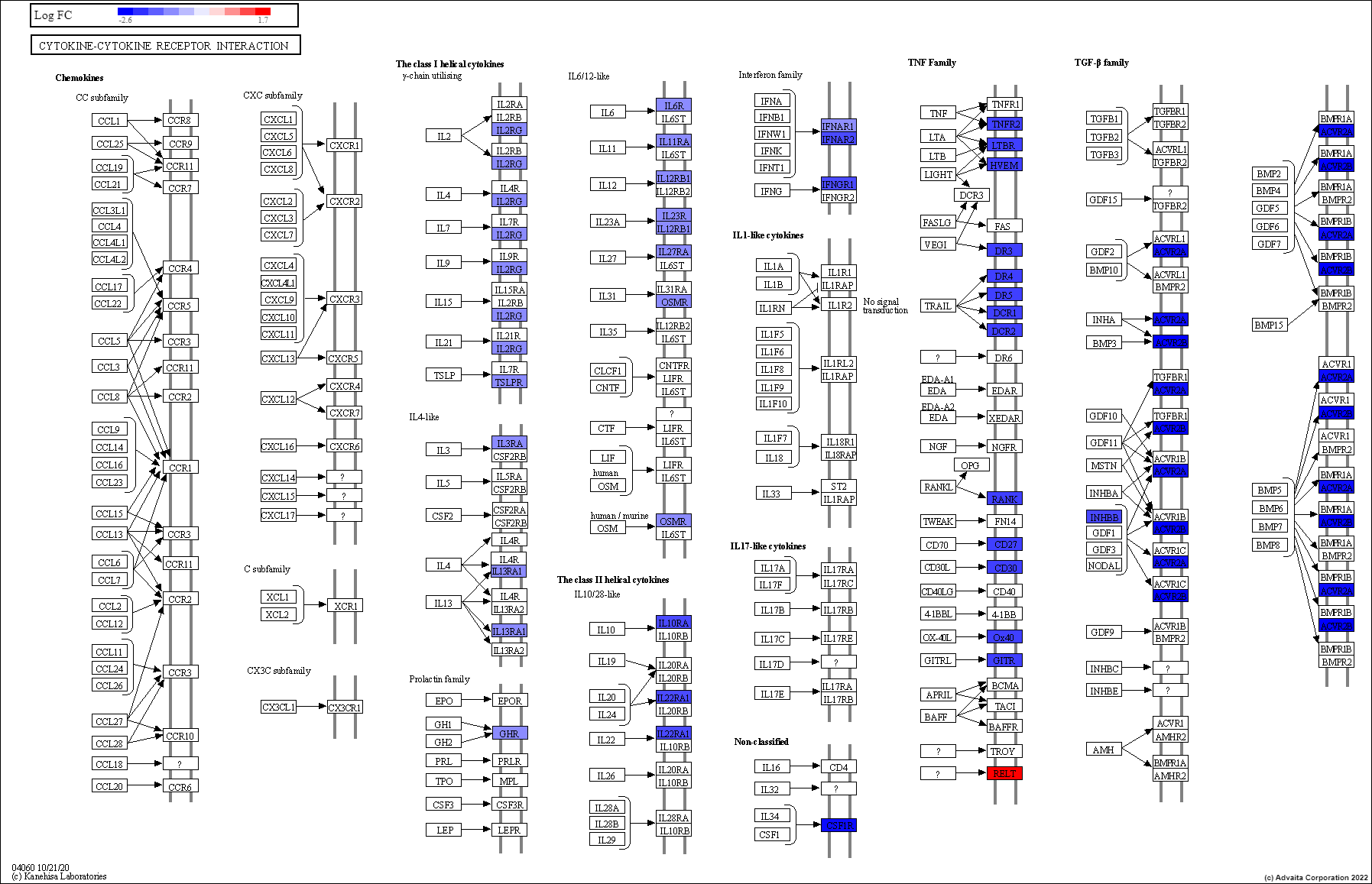

Supplement: Supplementary file 1 [file cells-12-01366-s001.zip › Supplementary_Figure_S6_IPG_Cytokine_cytokine_receptor_interaction.png]

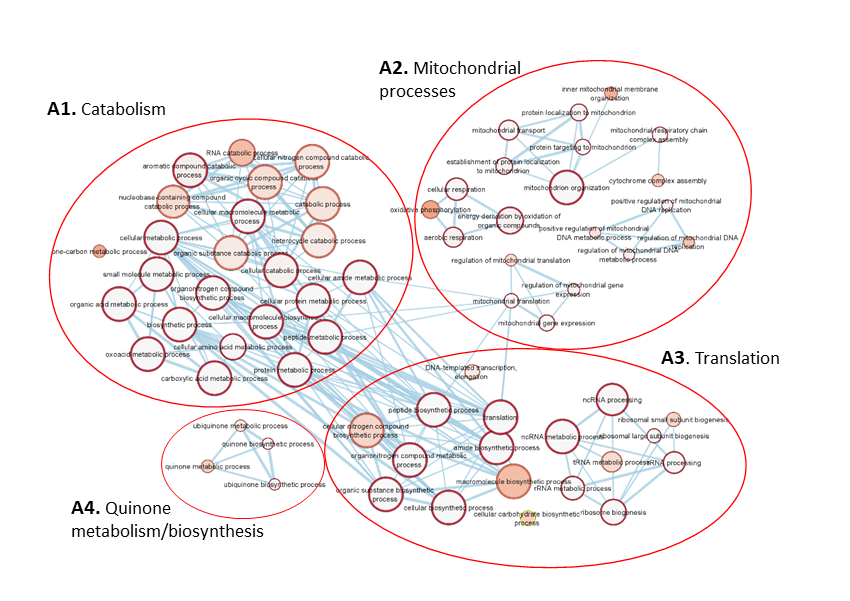

Supplement: Supplementary file 1 [file cells-12-01366-s001.zip › Supplementary_Figure_S7_Enrichmentmap_of_upregulated_genes_torpor+radiation_vs_control.tif]

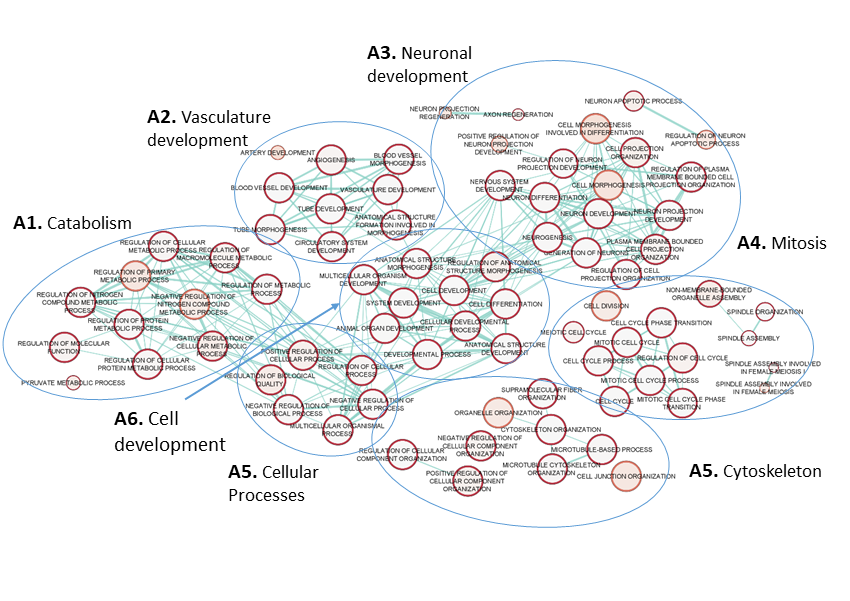

Supplement: Supplementary file 1 [file cells-12-01366-s001.zip › Supplementary_Figure_S8_Enrichmentmap_of_downregulated_genes_torpor+radiation_vs_control.tif]

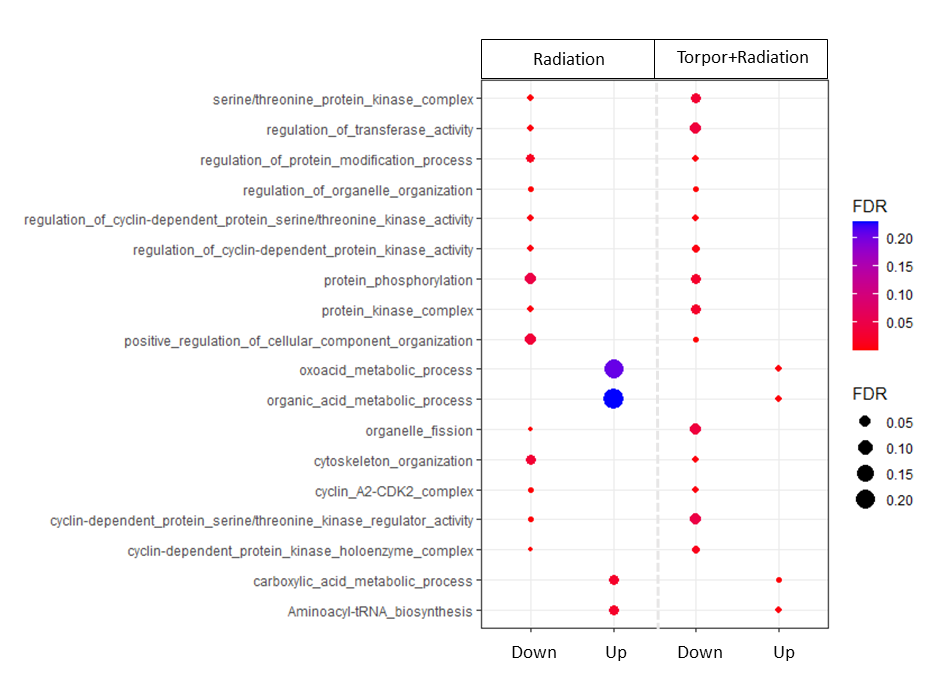

Supplement: Supplementary file 1 [file cells-12-01366-s001.zip › Supplementary_Figure_S9_Bubble_plot_showing_shared_GO_terms_in_torpor_radiation_vs_radiation_group.tif]

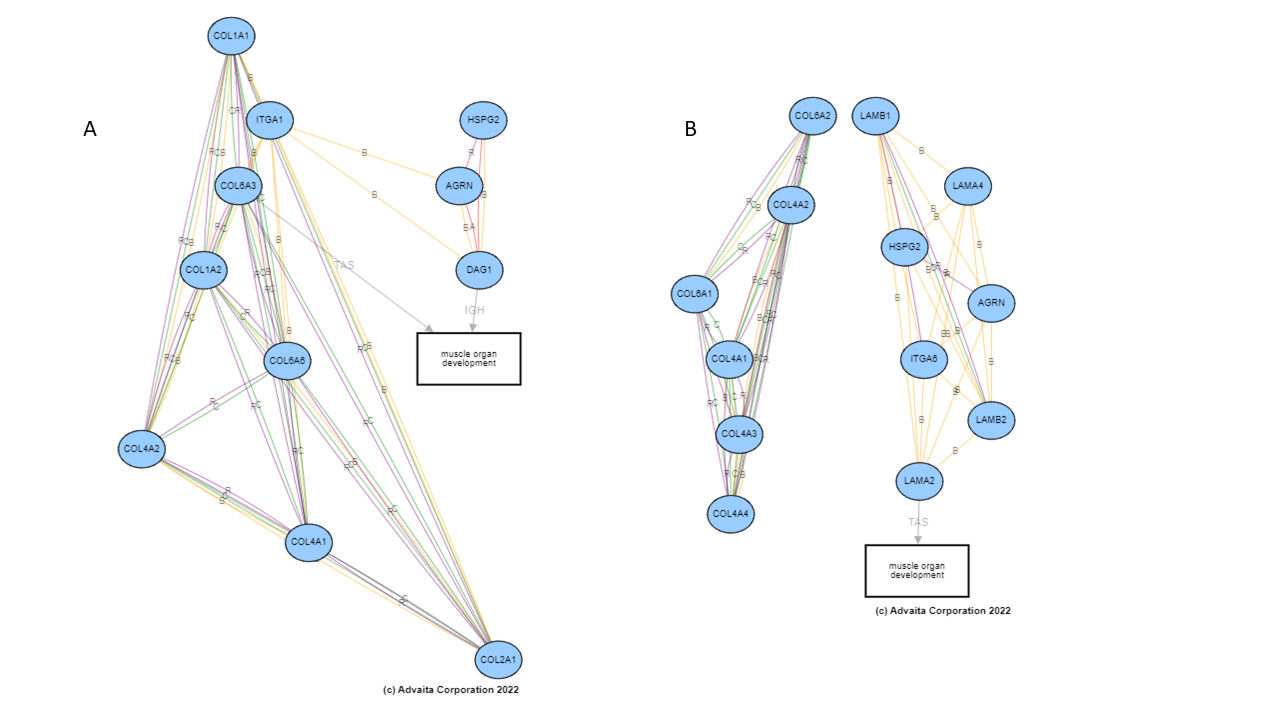

Supplement: Supplementary file 1 [file cells-12-01366-s001.zip › Supplementary_Figure_S10_ECM_development_network_analysis.tif]
